# Maternal plasma pro-atrial and C-type natriuretic peptide levels and their associations with cardiovascular and renal function in the second half of normal pregnancy: a longitudinal study

**DOI:** 10.1186/s12884-021-03824-3

**Published:** 2021-05-05

**Authors:** Rima D. Yarlagadda, Jonas Johnson, Åse Vårtun, Kari Flo, Ganesh Acharya

**Affiliations:** 1 Department of Clinical Medicine, Women’s Health and Perinatology Research Group, UiT-The Arctic University of Norway, Tromsø, Norway; 2 The University of Virginia, College of Arts and Science, Charlottesville, VA USA; 3 Division of Obstetrics and Gynecology, CLINTEC, Karolinska Institutet and Karolinska University Hospital, Stockholm, Sweden; 4 University Hospital of North Norway, Tromsø, Norway; 5 Department of Obstetrics and Gynecology, Akershus University Hospital, Lørenskog, Norway

**Keywords:** Cardiac function, Endothelial function, Natriuretic peptides, Pregnancy, Renal function

## Abstract

**Background:**

Physiological adaptation in pregnancy is characterized by remodeling of endocrine, cardiovascular and renal functions leading to fluid retention, volume expansion, altered cardiac loading conditions and hyperdynamic circulation. Natriuretic peptides have been used as biomarkers of cardiovascular function, but their associations with gestational age-related changes in maternal cardiac, endothelial and renal function have not been elucidated. The aim of this study was to establish longitudinal reference values for maternal plasma atrial natriuretic peptide (proANP) and C-type natriuretic peptide (CNP) and investigate their temporal association with cardiovascular and renal function in the second half of pregnancy.

**Methods:**

This study was a prospective longitudinal study of 53 low-risk pregnancies. Women were examined every 3–5 weeks during 22–40 weeks of gestation (252 observations). Fasting maternal blood samples were obtained to measure proANP, CNP, creatinine, cystatin C, uric acid, and fibrinogen levels. Cardiac function and systemic hemodynamics were assessed noninvasively by impedance cardiography (ICG) and vascular endothelial function by flow-mediated vasodilation of brachial artery (FMD).

**Results:**

The plasma proANP (R^2^adj = 0.79; *P* = 0.007), CNP (R2adj = 0.54; *P* = 0.005) decreased between 22 and 40 weeks. The creatinine (R^2^adj = 0.90; *P* < 0.001), cystatin C (R^2^adj = 0.93; P = < 0.001) and uric acid (R^2^adj = 0.83; *P* < 0.001) increased significantly, whereas the estimated glomerular filtration rate (R^2^adj = 0.93; *P* < 0.001) decreased with gestational age. The FMD did not change significantly but fibrinogen (R^2^adj = 0.79; *P* < 0.001) increased with advancing gestation. The maternal systemic vascular resistance index (R^2^adj = 0.50; *P* < 0.001) increased, stroke index (R^2^adj = 0.62; *P* < 0.001) decreased, whereas the cardiac index (R^2^adj = 0.62; *P* = 0.438) and thoracic fluid content (R^2^adj = 0.72; *P* = 0.132) did not change significantly with gestation. The proANP was associated with thoracic fluid content (R^2^adj = 0.74; *P* < 0.001) and fibrinogen (R^2^adj = 0.78; *P* = 0.034) but not with other variables of systemic hemodynamics, endothelial function, or renal function. The CNP was not associated significantly with parameters of cardiovascular or renal function.

**Conclusion:**

Longitudinal reference values for maternal plasma proANP and CNP were established. These natriuretic peptides decreased slightly with advancing gestation, but they did not reflect the temporal physiological changes in maternal systemic hemodynamics, vascular endothelial function and renal function during the second half of pregnancy. The proANP correlated with the thoracic fluid content reflecting volume load in pregnancy.

## Background

Natriuretic peptides play an important role in the regulation of body fluid-electrolyte homeostasis and cardiovascular function through their effect on diuresis, natriuresis and vascular tone [1–3]. Three types of natriuretic peptides that are commonly used as biomarkers in clinical practice are: atrial natriuretic peptide (ANP), brain natriuretic peptide (BNP), and C-type natriuretic peptide (CNP). The ANP and BNP are cardiac peptide hormones produced primarily by the atrium and ventricles, respectively [1], whereas CNP is expressed in a variety of tissues, particularly in high concentration in vascular endothelium, and acts locally in a paracrine/autocrine manner [3, 4]. ANP, BNP and CNP essentially share a similar spectrum of biological actions although their potencies may vary [5].

The ANP secretion is primarily stimulated by atrial stretch and enhanced under the conditions of increased intravascular volume load [6]. It is a volume regulating hormone that causes increased natriuresis by enhancing excretion of salt and water by kidneys [7]. CNP has no natriuretic activity, but it plays a key role in regulating vascular homeostasis [8]. Both, ANP and CNP, promote vasodilation and have blood pressure lowering effect. Pregnancy is a physiological state of hypervolemia, hyperdynamic circulation and altered vascular tone [9, 10]. Therefore, these natriuretic peptides are likely to have a fundamental role in fluid-electrolyte homeostasis and cardiovascular remodeling during pregnancy.

In human pregnancies, maternal plasma ANP levels have been reported to be similar or higher compared with non-pregnant state [11]. However, longitudinal studies have shown a decrease in maternal ANP levels after mid-pregnancy towards term in uncomplicated pregnancies ([12–15]. In line with several published studies, a more recent study with reasonably large sample size has demonstrated that maternal plasma BNP and NT-ProBNP levels remain stable throughout the pregnancy and are similar to that in nonpregnant state [16]. Maternal plasma CNP is reduced in healthy pregnant women compared to non-pregnant women, and levels tend to fall with advancing gestation [17, 18]. In a cross-sectional study we found that that proANP correlated negatively with maternal cardiac output and left ventricular cardiac work index, whereas the CNP did not correlate with any of the variables of cardiac function, systemic hemodynamics, or preload reserve in women with uncomplicated pregnancies at 22–24 weeks [19]. However, the role of these natriuretic peptides in the remodeling of cardiac, endothelial, and renal function during pregnancy has not been elucidated.

We hypothesized that maternal plasma proANP and CNP levels vary with gestational age, and there is an association between these natriuretic peptides and pregnancy-related physiological changes in cardiovascular and renal function. Our aim was to obtain serial measurements of plasma proANP and CNP in the second half of pregnancy in order to establish gestational age specific longitudinal reference values and determine if their levels correlate with temporal changes in maternal systemic hemodynamics, renal function and vascular endothelial function.

## Methods

### Study design

This study was part of a larger prospective study investigating maternal cardiovascular function in the second half of pregnancy approved by the Regional Committee for Medical and Health Research Ethics – North Norway (REK Nord 5.2005.1386). Low risk healthy pregnant women were recruited to this longitudinal arm of the study. Some data on maternal systemic hemodynamics, uterine artery blood flow and endothelial function from this cohort has been reported previously [20, 21]. Here we address gestational age related variations in plasma natriuretic peptide (proANP and CNP) levels in association with serial changes in maternal cardiac, vascular and renal function during the second half of pregnancy. Pregnant women were informed about the study by a midwife during their antenatal visit to the hospital for a routine second trimester ultrasound scan and assessed eligibility to participate in the study. The inclusion criteria were: age ≥ 18 years, no complications in the current pregnancy, and the ultrasound examination showing a singleton pregnancy without any major fetal or placental abnormality. The exclusion criteria were: multiple gestation, prior history of obstetric complications (e.g. pregnancy induced hypertension, preeclampsia, gestational diabetes, fetal growth restriction, preterm delivery before 34 weeks) or history of cardiovascular, endocrine, renal, liver or autoimmue diseases and use of any regular medication or smoking during pregnancy. If the woman met the inclusion criteria and expressed desire to participate in the study, a written informed consent was obtained, and research appointments were scheduled at approximately four-weekly intervals during 22–40 weeks of pregnancy. Women continued their routine antenatal care in parallel with these appointments. All measurements were performed between 08:00 and 10:00 h in a quiet room with the temperature maintained at around 22 °C after at least 8 h of overnight fasting.

### Anthropometry

The height of the pregnant woman was measured at the first visit using an altimeter (Charder Electronic Co, Taichung City, Taiwan) and body weight was determined using an electronic scale (Soehnle, Leifheit AG, Nassau, Germany) in light clothing without wearing shoes. The body surface areas (BSA) were calculated using DuBois formula [22] as: BSA = 0.007184*height^0.725^*weight^0.425^. This formula is validated in pregnant women [23].

### Collection of blood samples

Fasting venous blood samples (10 mL) were obtained from the antecubital vein and collected in two separate tubes containing ethylenediaminetetraacetic acid (EDTA) and sodium citrate, respectively using a vacutainer while the woman was resting in a supine semi-recumbent position. Plasma was separated from the blood cells by centrifugation at room temperature for 10 min at 1250 g, transferred to cryotube (Sigma Aldrich GmbH, Munich, Germany) and stored at − 70 °C until further analysis was performed. A single investigator (ÅV) performed all blood sampling, processing, and storage. The samples were analyzed in a single batch in the clinical biochemistry laboratory of University Hospital of North Norway, Tromsø, Norway. The laboratory participated in an external quality assessment program provided by Labquality (www.labquality.fi).

### Measurement of natriuretic peptides (proANP and CNP)

The proANP and CNP levels were quantified in maternal plasma samples using commercially available Enzyme-linked Immuno-sorbent assay (ELISA) kits according to the manufacturer’s instructions. The intra-assay and inter-assay coefficients of variation (CV) for the ELISA kit (EIA-4703, DRG, instruments, GmbH, Germany) used for the quantification of proANP were ≤ 5% and ≤ 9%, respectively and the lower limit of detection (LOD) expressed as value lying three standard deviations (SD) above that of lowest measurable analyte level was 0.05 nmol/L. The human C-type natriuretic peptide quantification kit (CSB-E08909h, CUSABIO, Lab-Tech AS, Norway) had an inter-assay CV of ≤10% and an intra-assay CV of ≤8%, and LOD was 15.6 pg/mL.

### Evaluation of renal function

#### Measurement of creatinine

Analysis of plasma creatinine was performed with an enzymatic in vitro assay standardized against isotope-dilution mass spectrometry (CREAplus, Cobas® Roche Diagnostics GmbH, Mannheim, Germany) using a Cobas® Modular P800 analyzer (Roche Diagnostics GmbH, Mannheim, Germany) that has an intra-assay CV of the assay was 1.0%, inter-assay CV 1.9% and the LOD was 0.10 mg/dL (8.84 μmol/L). The analysis at our laboratory has been calibrated with serum X in the Nordic Reference Interval Project.

#### Measurement of cystatin C

Plasma Cystatin C was measured with a particle-enhanced turbidimetric immunoassay (Gentian cystatin C immunoassay, Gentian AS, Moss, Norway) and a Cobas® Modular P800 analyzer (Roche Diagnostics GmbH, Mannheim, Germany) that has an intra-assay CV of 1.7%, inter-assay CV was 2.8% and LOD 0.40 mg/L. The inter-assay CV for internal quality control with pooled blood donor sera was 3.2%, Equalis AB (www.equalis.se) provided the external quality control.

#### Estimation of glomerular filtration rate

Estimated glomerular filtration rate *(eGFR)* was calculated using three different equations based on plasma cystatin C values as follows:

- eGFR (arbitrary units) = 100/Cystatin C (mg/L) [24]
- eGFR = 79.901 * Cystatin C (mg/L)^–1.4389^ [25]
- eGFR (mL/min/1.73m^2^) = 86.49 * Cystatin C (mg/L)^− 1.686^ * 0.948 [26, 27]

#### Measurement of uric acid

Plasma uric acid was measured using Roche uric acid plus assay and Cobas® Modular P800 analyzer (Roche Diagnostics GmbH, Mannheim, Germany) according to manufacturer’s instructions. The assay had a within-run CV of 0.5% and between-run CV of 1.7%, LOD of 11.9 μmol/L.

### Assessment of cardiac function and systemic hemodynamics

Maternal cardiac function and systemic hemodynamics was assessed with noninvasive impedance cardiography (ICG) using a commercially available system (ICG; Philips Medical Systems, Böblingen, Germany). The measurements were obtained after 15 min of rest with the participant lying quietly in a supine semi-recumbent position as described previously [20]. A single operator (ÅV) was designated to perform the ICG. The ICG equipment was connected to two pairs of dual sensing electrodes placed on each side of neck and two pairs on each side of the chest at mid-axillary line that continuously record the change in bioimpedance resulting from aortic velocity and volume change in time together with the ECG signals. A sphygmomanometer cuff wrapped around the left upper arm was also connected to automatically measure mean arterial blood pressure (MAP). Pregnant woman’s height, weight, and age were registered into the ICG system. Central venous pressure (CVP) was preset at 4 mmHg and pulmonary artery occlusion pressure (PAOP) at 8 mmHg. The following variables were directly measured by the ICG system based on impedance signals in conjunction with ECG signals: thoracic fluid content (TFC) that reflects the baseline electrical conductivity (1/Z_0_) of the thoracic cavity which is determined by total intravascular, alveolar and interstitial fluid in the thorax, pulsatile impedance (ΔZ) that reflects the blood volume ejected in systole, heart rate (HR), pre-ejection period (PEP), left ventricular ejection time (LVET), velocity index (VI) that represents the aortic peak blood flow velocity, and acceleration index (ACI) that reflects rate of aortic blood flow acceleration immediately after the opening of the aortic valve. Stroke volume (SV) was derived as: volume of electrically participating tissue*LVET*ejection phase contractility index (i.e. dZ/dt_max_*TFC). The following variables were calculated: stroke index (SI) = SV/BSA; cardiac index (CI) = (SV*HR)/BSA; systemic vascular resistance index (SVRI) = [(MAP-CVP)*80]/CI, and systolic time ratio (STR) = PEP/LVET.

### Assessment of vascular function

#### Flow-mediated vasodilation of brachial artery (FMD)

Maternal nitric oxide (NO) dependent vascular endothelial function was assessed by post-ischemic hyperemia-induced FMD as described previously [21] following the International Brachial Artery Reactivity Task Force guidelines [28]. A single operator (KF) performed all FMD measurements using an Acuson Sequoia 512, ultrasound system (Mountain View, CA, USA) equipped with an 8-MHz linear transducer. Electrocardiogram (ECG) signals were displayed continuously on the ultrasound screen. An inflatable pressure cuff was wrapped around the right upper arm with the lower boarder of the cuff about 8 cm proximal to the elbow. Brachial artery was identified by two-dimensional B-mode ultrasound imaging approximately 5 cm above the antecubital fossa and presence of flow was confirmed by color Doppler. Brachial artery blood flow velocity waveforms were recorded using spectral pulsed-wave Doppler and time-averaged maximum velocity (TAMxV) was measured automatically using the software of the ultrasound machine. The inner diameter of brachial artery (intima to intima) was measured in a magnified B-mode grey scale ultrasound image in the end-systole incident with the peak T-wave of the ECG. After the baseline measurements the cuff was inflated to occlude the brachial artery with a pressure at least 50 mmHg above the systolic blood pressure measured in the left arm during ICG measurement. The ischemia was maintained for 5 min, and the cuff was deflated. The measurements of the brachial artery TAMxV and diameter were repeated 15 s and diameter 60 s, respectively after the cuff deflation. The FMD was calculated as percentage change in the brachial artery diameter from the baseline following the release of the cuff: FMD% = (post-ischemic diameter – baseline diameter)/baseline diameter*100. The brachial artery blood flow (Q_BA_) was calculated as: Q_BA_ (mL/min) = TAMxV, cm/s*π (brachial artery diameter, cm/2)^2^*60. Post-ischemic reactive hyperemia was quantified as percentage increase in Q_BA_ from the baseline value as: (post-ischemic Q_BA_ – baseline Q_BA_)/ baseline Q_BA_*100.

#### Measurement of plasma fibrinogen

Fibrinogen was measured in citrate plasma using STA®-Fibrinogen reagent kit and STA-R analyzer (Diagnostica Stago Inc., Parsippany, NJ, USA) using the Clauss clotting method [29]. The test had an intra-assay CV of 1.3–3.4% and inter-assay CV of 2.0–3.7%, and LOD of 0.3 g/L.

### Statistical analyses

Data analysis was performed using MATLAB R2020b (Mathworks, Inc. Natick, MA). The sample size of the study was estimated based on the assumption that approximately 10–12 observations per gestational week between 22 and 40 weeks would be sufficient to construct reference intervals with adequate precision. We aimed to have a minimum of 200 observations to account for failure to obtain blood samples and/or ICG and FMD measurements. Categorical variables were presented as n (%) and continuous variables as mean (±SD) or median (range) as appropriate. Statistical significance was set to *P* < 0.05.

The assumption of normality was checked for each variable and logarithmic or power transformations were performed as required to achieve normal distribution based on Box-Cox transformation lambda (λ) value. Trend analysis for any significant association between a measured variable and the gestational age was done using the mean vector from the mixed model accounting for possible dependency between repeated measurements performed on the same women at different time points in pregnancy. The best fitting fractional polynomials were chosen from a list of 44 regression models to construct mean curves of measured variables in relation to gestational age [30]. The means and percentiles for each gestational week were calculated using multilevel modeling accounting for the longitudinal study design [31]. The individual observations were fitted as a linear function of the fractional polynomial term of the gestational age. A random intercept term was included for each individual and a random slope for the fractional polynomial term. The gestational age specific reference percentiles were initially calculated on the transformed scale, then appropriate reverse transformations were performed to obtain the values in the primary measurement scales. The linear associations between measured variables were tested using multilevel modeling considering the longitudinal design of the study. The gestational age specific z-scores were calculated as the z = (x-μ)/σ, where x is the individual value, μ is the mean value and σ is the standard deviation for a given gestational age.

## Results

A total of 53 participants completed the study with 252 observations available for analyses. The follow up was complete with the outcome data available for all the participants, and none of the women was excluded from the final data analysis.

### Demographic and clinical characteristics of the study participants

The participants’ median age was 28 years (range, 18–39), the mean body mass index at 23.5 ± 3.1 kg/m^2^, and the mean arterial pressure at booking was 83 ± 8 mmHg. Thirty-six (68%) women were nulliparous. There were no significant maternal or fetal complications during pregnancy. Three women were delivered by cesarean section (one for breech presentation and two due to slow progress of labor). All neonates were healthy at birth with a mean birthweight of 3562 ± 470 g, five-minute Apgar score of 10 (range 7–10), and mean umbilical artery pH 7.24 ± 0.10.

### Longitudinal changes in maternal plasma natriuretic peptide levels

**Fig. 1:**
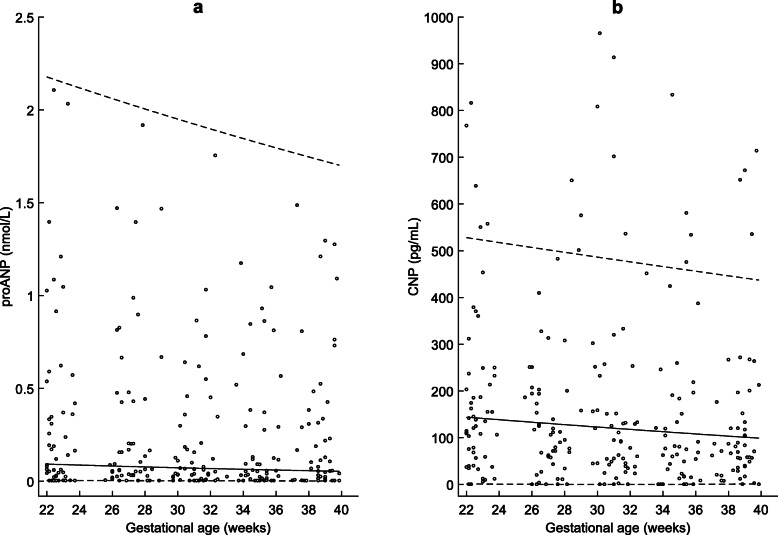
Maternal natriuretic peptides: Longitudinal changes in maternal plasma **a**) proAtrial natriuretic peptide (proANP) and **b**) C-type natriuretic peptide (CNP) levels in the second half of pregnancy (22–40 weeks of gestational age). The solid line represents the mean and dotted lines indicate 5th and 95th percentiles

Gestational age specific percentiles of maternal plasma proANP and CNP are presented in Table 1 and Table 2 and their corresponding reference charts with lines representing 5th, 50th and 95th percentiles are presented in Fig. 1. The mean plasma proANP (R^2^adj = 0.79; *P* = 0.007), CNP (R^2^adj = 0.54; *P* = 0.005) decreased slightly but significantly between 22 and 40 weeks.

**Table 1:** Percentiles of maternal plasma proAtrial natriuretic peptide (nmol/L) at 22–40 weeks of gestational age (GA)

		Percentile						
GA (weeks)	n	2.5th	5th	10th	50th	90th	95th	97.5th
22	35	0.002	0.004	0.008	0.093	1.085	2.178	3.987
23	15	0.002	0.004	0.008	0.090	1.066	2.148	3.945
24	0	0.002	0.004	0.007	0.087	1.048	2.119	3.903
25	2	0.002	0.003	0.007	0.085	1.030	2.090	3.862
26	20	0.002	0.003	0.007	0.082	1.012	2.061	3.821
27	19	0.002	0.003	0.006	0.080	0.994	2.033	3.781
28	9	0.002	0.003	0.006	0.077	0.977	2.005	3.741
29	7	0.002	0.003	0.006	0.075	0.960	1.977	3.702
30	15	0.001	0.003	0.006	0.073	0.943	1.950	3.663
31	23	0.001	0.003	0.005	0.070	0.927	1.924	3.624
32	7	0.001	0.002	0.005	0.068	0.911	1.897	3.586
33	5	0.001	0.002	0.005	0.066	0.895	1.871	3.548
34	17	0.001	0.002	0.005	0.064	0.880	1.846	3.511
35	19	0.001	0.002	0.004	0.062	0.865	1.820	3.474
36	5	0.001	0.002	0.004	0.060	0.850	1.796	3.437
37	9	0.001	0.002	0.004	0.059	0.835	1.771	3.401
38	18	0.001	0.002	0.004	0.057	0.820	1.747	3.365
39	22	0.001	0.002	0.004	0.055	0.806	1.723	3.330

**Table 2:** Percentiles of maternal plasma C-type natriuretic peptide (pg/mL) at 22–40 weeks of gestational age (GA)

		Percentile						
GA (weeks)	n	2.5th	5th	10th	50th	90th	95th	97.5th
22	35	1.19	1.02	11.81	143.92	422.60	528.13	629.23
23	16	1.45	0.81	11.04	141.19	417.86	522.82	623.42
24	0	1.73	0.62	10.30	138.48	413.15	517.53	617.63
25	2	2.04	0.45	9.58	135.80	408.47	512.28	611.88
26	19	2.38	0.31	8.90	133.14	403.81	507.05	606.15
27	19	2.74	0.20	8.23	130.51	399.18	501.84	600.44
28	9	3.13	0.11	7.59	127.91	394.58	496.67	594.77
29	7	3.54	0.05	6.98	125.34	390.00	491.52	589.12
30	14	3.98	0.01	6.40	122.79	385.45	486.39	583.49
31	23	4.44	0.00	5.84	120.26	380.93	481.30	577.90
32	7	4.93	0.01	5.30	117.77	376.43	476.23	572.33
33	5	5.44	0.05	4.79	115.29	371.96	471.19	566.79
34	16	5.98	0.12	4.31	112.85	367.52	466.17	561.27
35	18	6.54	0.21	3.85	110.43	363.10	461.18	555.79
36	5	7.13	0.33	3.42	108.04	358.71	456.22	550.32
37	8	7.75	0.47	3.01	105.67	354.35	451.28	544.89
38	19	8.39	0.64	2.63	103.33	350.01	446.38	539.48
39	23	9.06	0.83	2.28	101.02	345.70	441.49	534.10

### Longitudinal changes in maternal renal function

**Fig. 2:**
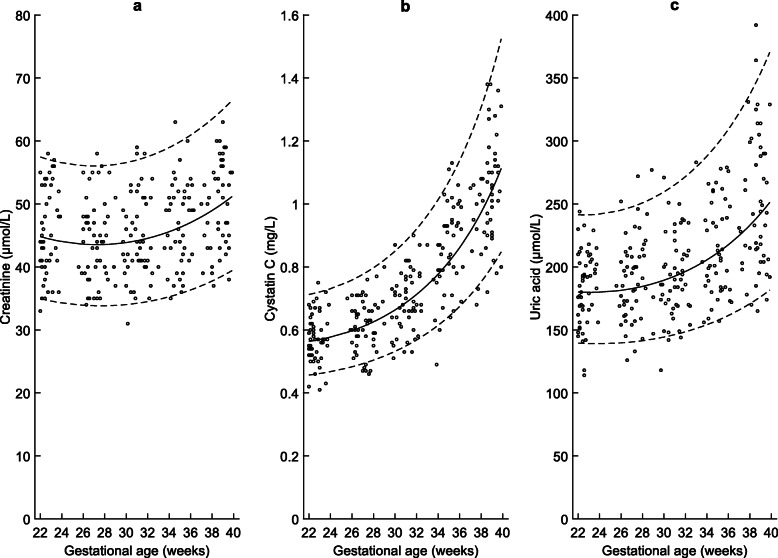
Biochemical markers of maternal renal function: Longitudinal changes in maternal plasma **a**) Creatinine, **b**) Cystatin C, and **c**) Uric acid levels in the second half of pregnancy (22–40 weeks of gestational age). The solid line represents the mean and dotted lines indicate 5th and 95th percentiles

**Fig. 3:**
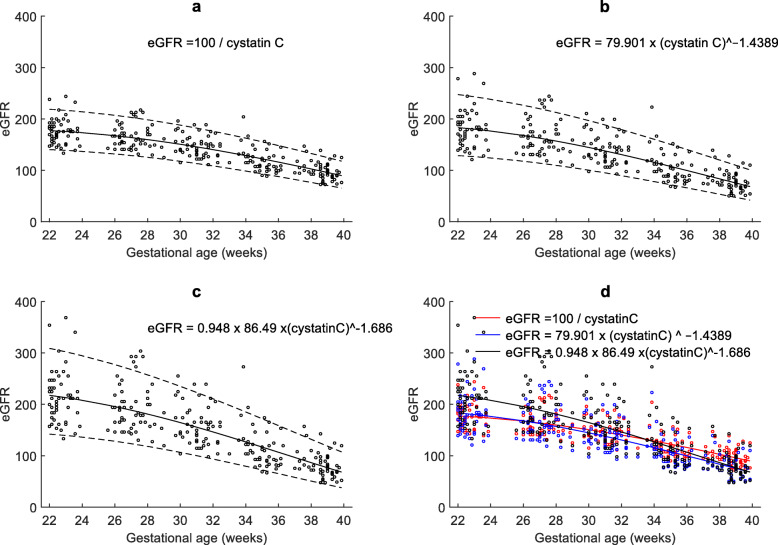
Maternal estimated glomerular filtration: Longitudinal changes in maternal cystatin C based estimated glomerular filtration rate (ml/min/1.73m^2^) calculated using three different equations, **a**) Perkins **b**) Jonsson **c**) Grubb, and **d**) their comparison in the second half of pregnancy (22–40 weeks of gestational age). The solid line represents the mean and dotted lines indicate 5th and 95th percentiles

Gestational age specific percentiles of maternal serum creatinine, cystatin C, and uric acid and are presented in Tables 3, 4, 5 and that of cystatin C based eGFR calculated using three different equations are presented in Tables 6, 7, 8. Their corresponding reference charts with lines representing 5th, 50th and 95th percentiles are presented in Figs. 2 and 3. The mean plasma creatinine (R^2^adj = 0.90; *P* < 0.001), cystatin C (R^2^adj = 0.93; *P* = < 0.001) and uric acid (R^2^adj = 0.83; *P* < 0.001) increased significantly from 22 to 40 weeks. The eGFR decreased significantly with gestational age (R^2^adj = 0.93; *P* < 0.001). The mean eGFR values were generally ≥ 120 mL/min/1.73m^2^ before 30 weeks of gestation irrespective of which of the three equations were used for its calculation, but after 30 weeks, 29.1, 40.0 and 33.7% of the values were < 120 using Perkins, Jonsson and Grubb equations, respectively.

**Table 3:** Percentiles of maternal plasma Creatinine (μmol/L) at 22–40 weeks of gestational age (GA)

		Percentile						
GA (weeks)	n	2.5th	5th	10th	50th	90th	95th	97.5th
22	36	33.33	34.95	36.92	44.82	54.40	57.46	60.26
23	16	32.98	34.59	36.54	44.38	53.89	56.94	59.73
24	0	32.69	34.30	36.24	44.04	53.51	56.54	59.31
25	2	32.48	34.08	36.01	43.78	53.23	56.26	59.02
26	20	32.33	33.93	35.86	43.62	53.06	56.09	58.86
27	19	32.25	33.85	35.79	43.55	53.01	56.04	58.81
28	9	32.24	33.84	35.78	43.57	53.06	56.10	58.88
29	7	32.29	33.90	35.85	43.68	53.22	56.28	59.08
30	15	32.41	34.03	35.99	43.87	53.49	56.57	59.39
31	23	32.60	34.23	36.21	44.16	53.87	56.98	59.83
32	7	32.85	34.50	36.50	44.54	54.36	57.52	60.40
33	5	33.18	34.84	36.87	45.02	54.97	58.17	61.10
34	17	33.57	35.26	37.32	45.60	55.71	58.96	61.93
35	19	34.04	35.76	37.86	46.27	56.56	59.87	62.90
36	5	34.59	36.34	38.48	47.06	57.55	60.93	64.02
37	9	35.22	37.01	39.18	47.95	58.68	62.13	65.29
38	18	35.93	37.76	39.99	48.96	59.95	63.49	66.72
39	21	36.73	38.61	40.89	50.10	61.37	65.00	68.33

**Table 4:** Percentiles of maternal plasma Cystatin C (mg/L) at 22–40 weeks of gestational age (GA)

		Percentile						
GA (weeks)	n	2.5th	5th	10th	50th	90th	95th	97.5th
22	36	0.44	0.46	0.48	0.56	0.68	0.71	0.75
23	16	0.44	0.46	0.48	0.57	0.68	0.72	0.76
24	0	0.45	0.47	0.49	0.58	0.69	0.73	0.77
25	2	0.46	0.47	0.50	0.59	0.70	0.74	0.78
26	20	0.46	0.48	0.51	0.60	0.72	0.76	0.80
27	19	0.47	0.49	0.52	0.61	0.73	0.78	0.82
28	9	0.48	0.50	0.53	0.63	0.75	0.80	0.84
29	7	0.50	0.52	0.54	0.64	0.78	0.82	0.86
30	15	0.51	0.53	0.56	0.66	0.80	0.85	0.90
31	23	0.53	0.55	0.58	0.69	0.83	0.88	0.93
32	7	0.55	0.57	0.60	0.71	0.87	0.92	0.97
33	5	0.57	0.59	0.62	0.74	0.91	0.96	1.02
34	17	0.59	0.61	0.65	0.78	0.95	1.01	1.07
35	19	0.62	0.64	0.68	0.82	1.01	1.07	1.13
36	5	0.65	0.67	0.71	0.86	1.07	1.14	1.21
37	9	0.68	0.71	0.75	0.91	1.14	1.22	1.29
38	19	0.72	0.75	0.79	0.97	1.22	1.31	1.39
39	23	0.76	0.80	0.85	1.04	1.32	1.42	1.51

**Table 5:** Percentiles of maternal plasma Uric acid (μmol/L) at 22–40 weeks of gestational age (GA)

		Percentile						
GA (weeks)	n	2.5th	5th	10th	50th	90th	95th	97.5th
22	36	133.38	139.57	147.25	180.16	225.46	241.42	256.67
23	16	133.00	139.20	146.89	179.88	225.36	241.40	256.74
24	0	132.87	139.10	146.83	180.00	225.82	242.00	257.49
25	2	133.00	139.27	147.06	180.52	226.85	243.23	258.93
26	21	133.39	139.72	147.59	181.45	228.45	245.10	261.07
27	19	134.03	140.44	148.42	182.79	230.65	247.64	263.96
28	9	134.94	141.45	149.56	184.57	233.47	250.88	267.61
29	7	136.11	142.75	151.02	186.79	236.95	254.85	272.08
30	15	137.58	144.36	152.81	189.48	241.12	259.61	277.43
31	23	139.33	146.28	154.96	192.67	246.04	265.22	283.73
32	7	141.40	148.54	157.47	196.40	251.78	271.75	291.06
33	5	143.80	151.17	160.38	200.71	258.40	279.29	299.54
34	17	146.55	154.18	163.73	205.65	266.00	287.96	309.30
35	19	149.69	157.60	167.53	211.28	274.70	297.89	320.48
36	5	153.24	161.48	171.84	217.67	284.62	309.24	333.29
37	9	157.24	165.86	176.71	224.92	295.93	322.21	347.95
38	19	161.75	170.79	182.19	233.12	308.83	337.03	364.75
39	22	166.80	176.33	188.36	242.41	323.55	354.00	384.04

**Table 6:** Percentiles of Cystatin C based estimated glomerular filtration rate using Perkins equation at 22–40 weeks of gestational age (GA)

		Percentile						
GA (weeks)	n	2.5th	5th	10th	50th	90th	95th	97.5th
22	36	133.70	140.31	148.12	177.42	209.36	218.88	227.32
23	16	132.22	138.77	146.52	175.55	207.21	216.65	225.01
24	0	130.36	136.84	144.51	173.24	204.58	213.93	222.21
25	2	128.13	134.53	142.10	170.50	201.48	210.72	218.91
26	20	125.53	131.84	139.31	167.33	197.92	207.05	215.13
27	19	122.57	128.79	136.14	163.75	193.91	202.91	210.89
28	9	119.28	125.39	132.62	159.78	189.47	198.34	206.20
29	7	115.66	121.65	128.75	155.43	184.62	193.34	201.07
30	15	111.73	117.60	124.56	150.72	179.37	187.93	195.53
31	23	107.51	113.25	120.05	145.66	173.74	182.14	189.59
32	7	103.03	108.63	115.27	140.28	167.76	175.98	183.28
33	5	98.30	103.75	110.21	134.61	161.44	169.48	176.61
34	17	93.35	98.64	104.92	128.66	154.82	162.66	169.63
35	19	88.20	93.33	99.42	122.47	147.92	155.56	162.34
36	5	82.89	87.85	93.73	116.06	140.76	148.19	154.79
37	9	77.44	82.22	87.89	109.46	133.39	140.60	147.01
38	19	71.89	76.47	81.93	102.71	125.83	132.81	139.01
39	23	66.26	70.64	75.87	95.83	118.12	124.86	130.85

**Table 7:** Percentiles of Cystatin C based estimated glomerular filtration rate (mL/min/1.73m^2^) using Jonsson equation at 22–40 weeks of gestational age (GA)

		Percentile						
GA (weeks)	n	2.5th	5th	10th	50th	90th	95th	97.5th
22	36	119.28	128.64	139.86	183.23	232.45	247.45	260.85
23	16	117.40	126.58	137.59	180.15	228.43	243.14	256.27
24	0	115.08	124.07	134.85	176.52	223.80	238.20	251.06
25	2	112.32	121.11	131.64	172.37	218.58	232.66	245.23
26	20	109.15	117.72	127.99	167.71	212.80	226.54	238.81
27	19	105.58	113.91	123.91	162.57	206.48	219.86	231.82
28	9	101.64	109.73	119.43	156.97	199.65	212.66	224.28
29	7	97.35	105.17	114.57	150.95	192.33	204.96	216.25
30	15	92.74	100.29	109.36	144.52	184.58	196.81	207.74
31	23	87.84	95.10	103.83	137.73	176.41	188.23	198.79
32	7	82.68	89.65	98.03	130.61	167.87	179.26	189.46
33	5	77.30	83.96	91.98	123.21	159.00	169.96	179.77
34	17	71.74	78.08	85.72	115.56	149.85	160.36	169.78
35	19	66.04	72.05	79.31	107.71	140.45	150.51	159.53
36	5	60.24	65.91	72.77	99.71	130.88	140.47	149.07
37	9	54.39	59.72	66.17	91.60	121.17	130.29	138.47
38	19	48.54	53.51	59.55	83.45	111.37	120.01	127.77
39	23	42.74	47.35	52.97	75.31	101.56	109.71	117.04

**Table 8:** Percentiles of Estimated glomerular filtration rate (mL/min/1.73m^2^) using Grubb equation at 22–40 weeks of gestational age

		Percentile						
GA (weeks)	n	2.5th	5th	10th	50th	90th	95th	97.5th
22	36	129.51	142.12	157.38	217.49	287.31	308.84	328.15
23	16	127.13	139.45	154.34	213.00	281.09	302.08	320.91
24	0	124.22	136.21	150.72	207.84	274.11	294.54	312.86
25	2	120.78	132.44	146.54	202.02	266.40	286.25	304.04
26	20	116.86	128.16	141.82	195.60	258.00	277.24	294.50
27	19	112.47	123.39	136.59	188.59	248.95	267.56	284.26
28	9	107.65	118.17	130.89	181.03	239.29	257.26	273.38
29	7	102.43	112.53	124.75	172.98	229.06	246.37	261.90
30	15	96.84	106.51	118.22	164.46	218.32	234.95	249.88
31	23	90.94	100.16	111.34	155.54	207.12	223.06	237.37
32	7	84.76	93.52	104.15	146.26	195.51	210.75	224.44
33	5	78.36	86.64	96.71	136.69	183.56	198.09	211.14
34	17	71.78	79.58	89.07	126.87	171.34	185.14	197.55
35	19	65.09	72.40	81.30	116.88	158.90	171.97	183.73
36	5	58.34	65.15	73.46	106.79	146.33	158.66	169.76
37	9	51.61	57.90	65.61	96.66	133.70	145.28	155.72
38	19	44.95	50.73	57.82	86.56	121.09	131.92	141.69
39	23	38.44	43.70	50.17	76.59	108.59	118.66	127.76

### Longitudinal changes in maternal cardiac function, systemic hemodynamics

**Fig. 4:**
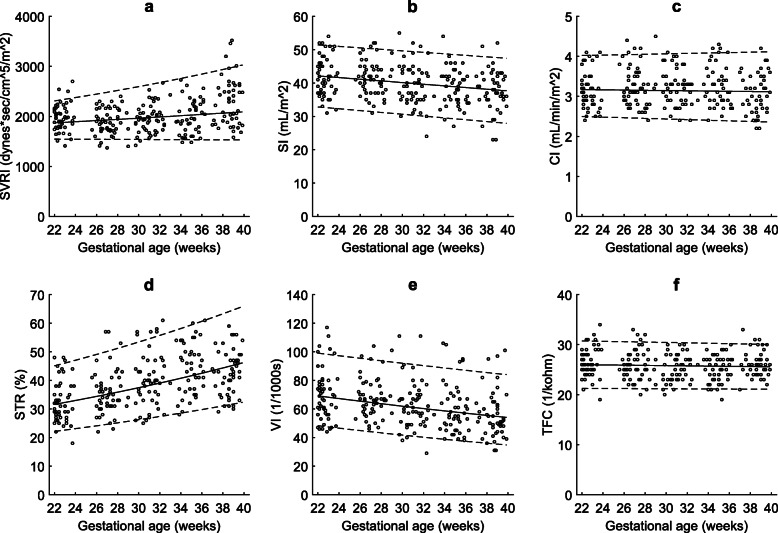
Maternal systemic hemodynamics: Longitudinal changes in maternal systemic hemodynamics and thoracic fluid content (TFC) in the second half of pregnancy (22–40 weeks of gestational age). The solid line represents the mean and dotted lines indicate 5th and 95th percentiles

Maternal heart rate (R^2^adj = 0.63; *P* < 0.001), systolic blood pressure (R^2^adj = 0.61; *P* = 0.006), diastolic blood pressures (R^2^adj = 0.73; *P* < 0.001) and MAP (R^2^adj = 0.70; *P* < 0.001) increased with gestation during the second half of pregnancy. The PEP (R^2^adj = 0.55; P < 0.001) increased while the LVET (R^2^adj = 0.50; *P* < 0.026) and ACI (R^2^adj = 0.72; *P* < 0.001) decreased. The SVRI (R^2^adj = 0.50; *P* < 0.001) and the STR (R^2^adj = 0.60; P < 0.001) increased, the SI (R^2^adj = 0.62; *P* < 0.001) and VI (R^2^adj = 0.69; *P* < 0.001) decreased, and the CI (R^2^adj = 0.62; *P* = 0.438) and TFC (R^2^adj = 0.72; *P* = 0.132) did not change significantly with advancing gestational age (Fig. 4).

### Longitudinal changes in maternal vascular function

**Fig. 5:**
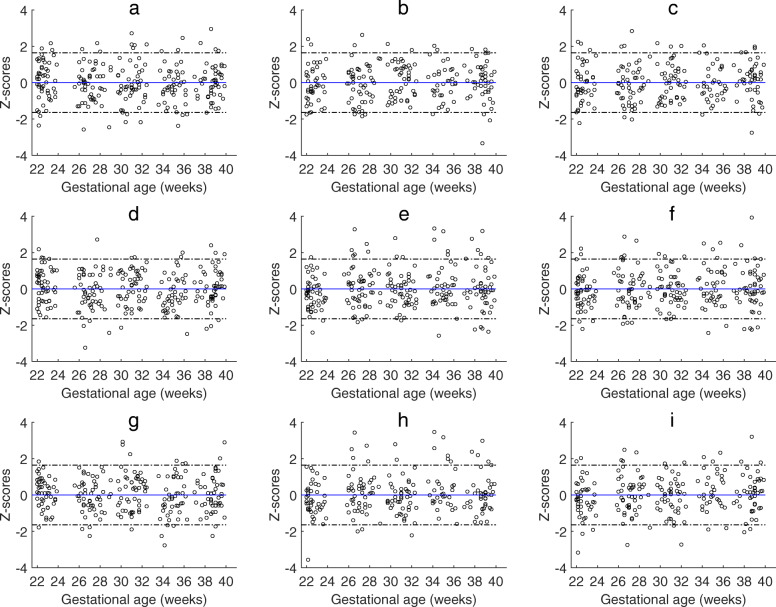
Maternal brachial artery blood flow: Gestational age specific z-scores (with 95% confidence limits) of maternal brachial artery baseline **a**) diameter, **b**) time-averaged maximum velocity (TAMxV) and blood flow (Q_BA_) and hyperemic (after release of occlusion) **e**) diameter, **f**) TAMxV and **g**) Q_BA,_ and the corresponding delta values of **h**) diameter, **i**) TAMxV and **j**) Q_BA_ in the second half of pregnancy (22–40 weeks of gestational age). The solid line represents the mean and dotted lines indicate 95% confidence intervals

**Fig. 6:**
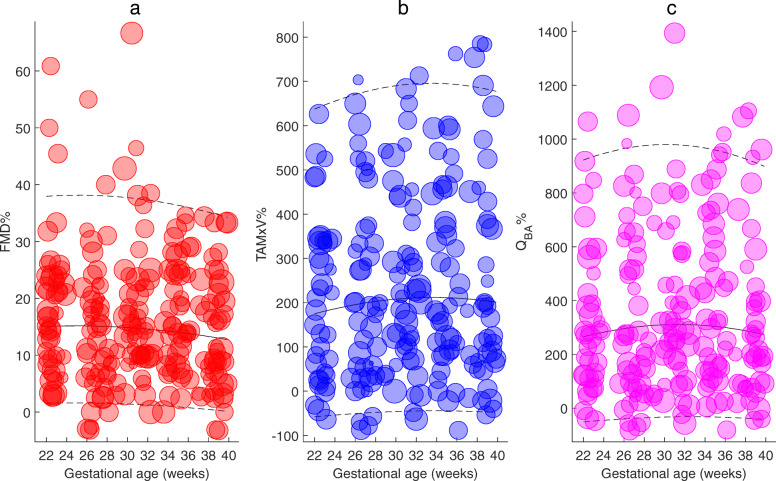
Maternal vascular endothelial function: Bubble chart showing longitudinal changes in brachial artery **a**) flow mediated vasodilation (FMD%) and **b**) percentage change in time averaged maximum velocity (TAMxV%) and **c**) blood flow (Q_BA_%) from baseline to post-ischemic hyperemia after the release of occlusion in the second half of pregnancy (22–40 weeks of gestational age). The size of the bubbles represents the baseline brachial artery diameter. The solid lines represent the means and dotted lines indicate 5th and 95th percentiles

Maternal NO-dependent vascular endothelial function assessed by brachial artery FMD and its relationship with inflammatory markers and uterine artery blood flow have been reported previously from this cohort [21]. A more detailed analyses performed in this study showed that the baseline brachial artery diameter (R^2^adj = 0.59; *P* < 0.001), TAMxV (R^2^adj = 0.28; *P* = 0.001), and Q_BA_ (R^2^adj = 0.40; P < 0.001) and post-ischemic brachial artery diameter (R^2^adj = 0.60; *P* < 0.001), TAMxV (R^2^adj = 0.07; *P* = 0.020), and Q_BA_ (R^2^adj = 0.012; *P* < 0.001) were significantly associated and increased with gestational age. The delta values of the brachial artery diameter (R^2^adj = 0.005; *P* = 0.532) and TAMxV (R^2^adj = 0.001; *P* = 0.267) did not change significantly, whereas the delta Q_BA_ (R^2^adj = 0.04; *P* = 0.006) increased significantly with gestation. The FMD% (R^2^adj = 0.00; *P* = 0.657), TAMxV% (R^2^adj = 0.07; *P* = 0.742) and Q_BA_% (R^2^adj = 0.10; *P* = 0.606) did not change significantly during 22–40 weeks of gestation. The z-scores of the brachial artery diameter, TAMxV, and Q_BA_ at baseline and after the release of pressure cuff following 5 min of occlusion, and the absolute differences between the corresponding two values (delta values) are presented in Fig. 5. The bubble charts demonstrating the percentage change in brachial artery diameter (FMD%), TAMxV%, and Q_BA_% are presented in Fig. 6. The FMD% was affected by the baseline brachial artery diameter with smaller caliber artery having higher FMD% as demonstrated by the bubble chart. Gestational age specific percentiles of brachial artery FMD% and Q_BA_% are presented in Tables 9 and 10.

**Table 9:** Percentiles of brachial artery Flow-mediated vasodilation (FMD%) at 22–40 weeks of gestational age (GA)

		Percentile						
GA (weeks)	n	2.5th	5th	10th	50th	90th	95th	97.5th
22	33	−1.00	0.86	3.26	14.49	31.69	38.04	44.22
23	15	−0.90	0.97	3.38	14.63	31.87	38.23	44.41
24	0	−0.82	1.06	3.47	14.75	32.00	38.37	44.55
25	2	−0.75	1.13	3.55	14.84	32.09	38.46	44.64
26	21	−0.69	1.19	3.61	14.90	32.14	38.49	44.66
27	19	−0.65	1.22	3.64	14.93	32.13	38.48	44.63
28	9	−0.63	1.25	3.66	14.93	32.09	38.41	44.54
29	7	−0.62	1.25	3.66	14.89	31.99	38.29	44.39
30	14	−0.63	1.23	3.64	14.83	31.85	38.12	44.19
31	23	−0.66	1.20	3.60	14.74	31.67	37.89	43.93
32	7	−0.70	1.15	3.54	14.62	31.44	37.62	43.61
33	3	−0.75	1.09	3.46	14.47	31.17	37.30	43.24
34	17	−0.83	1.00	3.36	14.29	30.85	36.93	42.82
35	19	−0.91	0.90	3.24	14.08	30.49	36.51	42.34
36	5	−1.01	0.79	3.10	13.84	30.09	36.05	41.81
37	8	−1.13	0.65	2.95	13.58	29.65	35.54	41.24
38	19	−1.26	0.51	2.78	13.29	29.17	34.98	40.61
39	22	−1.41	0.34	2.59	12.98	28.65	34.39	39.94

**Table 10:** Percentiles of percentage change in brachial artery blood flow from baseline to post-ischemic hyperemia (Q_BA_%) at 22–40 weeks of gestational age (GA)

		Percentile						
GA (weeks)	n	2.5th	5th	10th	50th	90th	95th	97.5th
22	26	−72.16	−49.87	−8.76	268.62	752.05	926.36	1091.01
23	14	−70.52	−47.07	−4.69	276.52	762.81	937.76	1102.90
24	0	−68.92	−44.44	−0.94	283.61	772.27	947.72	1113.22
25	2	−67.41	−42.02	2.46	289.86	780.41	956.21	1121.96
26	19	−66.01	−39.82	5.49	295.25	787.19	963.21	1129.09
27	18	−64.75	−37.89	8.13	299.76	792.61	968.71	1134.60
28	7	−63.65	−36.23	10.35	303.37	796.65	972.70	1138.49
29	7	−62.73	−34.87	12.15	306.07	799.31	975.17	1140.73
30	11	−61.99	−33.82	13.49	307.86	800.57	976.11	1141.33
31	21	−61.46	−33.09	14.39	308.71	800.43	975.52	1140.29
32	7	−61.14	−32.68	14.82	308.64	798.90	973.41	1137.60
33	3	−61.04	−32.61	14.79	307.63	795.98	969.77	1133.28
34	16	−61.15	−32.86	14.30	305.71	791.67	964.62	1127.34
35	16	−61.48	−33.45	13.35	302.86	785.99	957.96	1119.77
36	3	−62.02	−34.36	11.95	299.10	778.94	949.81	1110.61
37	6	−62.77	−35.58	10.10	294.45	770.55	940.19	1099.86
38	15	−63.70	−37.11	7.82	288.93	760.84	929.12	1087.55
39	18	−64.81	−38.92	5.13	282.54	749.82	916.63	1073.71

**Fig. 7:**
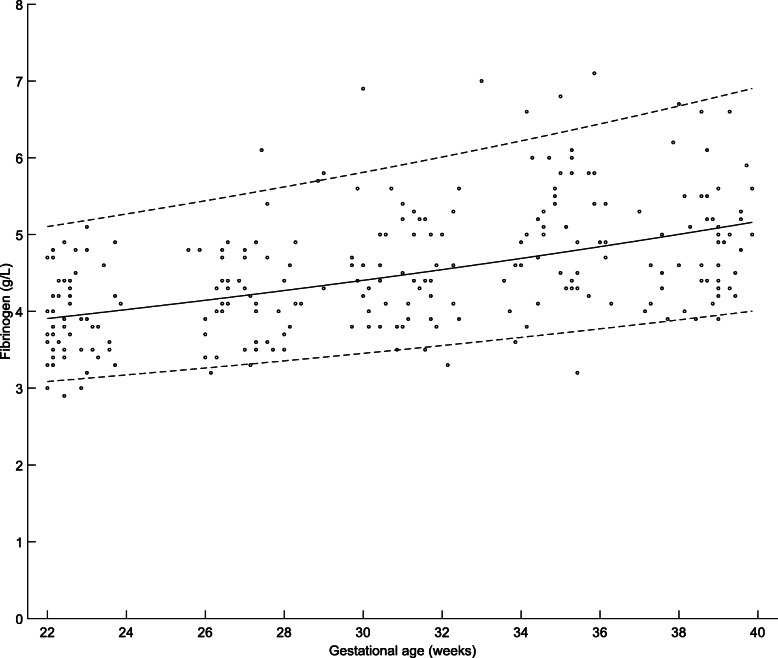
Maternal plasma Fibrinogen levels: Longitudinal changes in maternal plasma Fibrinogen levels in the second half of pregnancy (22–40 weeks of gestational age). The solid line represents the mean and dotted lines indicate 5th and 95th percentiles

Gestational age specific percentiles of maternal plasma fibrinogen during 22–40 gestational weeks are presented in Table 11. Fibrinogen levels increased significantly (R^2^adj = 0.79; *P* < 0.001**)** with advancing gestation (Fig. 7).

**Table 11:** Percentiles of maternal plasma Fibrinogen (g/L)) at 22–40 weeks of gestational age (GA)

		Percentile						
GA (weeks)	n	2.5th	5th	10th	50th	90th	95th	97.5th
22	35	2.96	3.09	3.24	3.91	4.80	5.10	5.40
23	16	3.00	3.13	3.29	3.96	4.87	5.18	5.48
24	0	3.04	3.17	3.33	4.02	4.95	5.27	5.57
25	2	3.08	3.22	3.38	4.08	5.03	5.35	5.66
26	20	3.13	3.26	3.43	4.14	5.11	5.44	5.76
27	18	3.17	3.31	3.48	4.21	5.19	5.53	5.85
28	9	3.21	3.36	3.53	4.27	5.27	5.62	5.95
29	7	3.26	3.40	3.58	4.34	5.36	5.71	6.05
30	15	3.31	3.45	3.63	4.40	5.45	5.81	6.16
31	23	3.35	3.50	3.69	4.47	5.54	5.91	6.26
32	7	3.40	3.55	3.74	4.54	5.63	6.01	6.37
33	5	3.45	3.61	3.80	4.62	5.73	6.11	6.48
34	16	3.50	3.66	3.86	4.69	5.82	6.22	6.60
35	19	3.56	3.72	3.92	4.76	5.92	6.33	6.72
36	5	3.61	3.77	3.98	4.84	6.03	6.44	6.84
37	9	3.66	3.83	4.04	4.92	6.13	6.56	6.96
38	19	3.72	3.89	4.10	5.00	6.24	6.67	7.09
39	23	3.78	3.95	4.16	5.09	6.35	6.80	7.22

### Association between natriuretic peptides and systemic hemodynamics

The plasma proANP was associated significantly with thoracic fluid content (R^2^adj = 0.74; *P* < 0.001), but not with other variables of maternal systemic hemodynamics assessed by ICG. The CNP was not associated significantly with any variables measured by ICG.

### Association between natriuretic peptides and vascular function

Both proANP and CNP were neither significantly associated with brachial artery FMD% (R^2^adj = 0.58; *P* = 0.286 and R^2^adj = 0.000; *P* = 0.927, respectively) nor with Q_BA_% (R^2^adj = 0.00; *P* = 0.351 and R^2^adj = 0.136; *P* = 0.176, respectively). Maternal proANP and fibrinogen correlated significantly **(**R^2^adj = 0.78; *P* = 0.034) but no significant correlation was observed between CNP and maternal fibrinogen levels (R^2^adj = 0.57; *P* = 0.101).

### Association between natriuretic peptides and renal function

The proANP was not associated significantly with plasma uric acid (R^2^adj = 0.78; *P* = 0.057), creatinine (R^2^adj = 0.78; *P* = 0.664), cystatin C (R^2^adj = 0.78; *P* = 0.086), or eGFR (R^2^adj = 0.06; *P* = 0.264). The CNP also did not correlate significantly with uric acid (R^2^adj = 0.55; *P* = 0.873), creatinine (R^2^adj = 0.55; *P* = 0.704), cystatin C (R^2^adj = 0.56; *P* = 0.090), or eGFR (R^2^adj = 0.05; *P* =*n* =0.416).

## Discussion

### Main findings

This study showed that maternal plasma proANP and CNP levels decrease slightly but significantly with advancing gestational age between 22 and 40 weeks. None of these natriuretic peptides correlated significantly with the maternal cardiac, hemodynamic, endothelial, or renal function. However, we found a strong positive correlation between proANP and the baseline conductivity of the thorax, a measure of total amount of intravascular and extravascular fluid in the thoracic cavity.

### Interpretation of results

Serial changes in maternal hemodynamics assessed by noninvasive ICG in this study reflected temporal physiological changes that are known to occur in the second half of normal pregnancy with significantly increasing heart rate, blood pressure and SVRI, and a stable CI. This is in line with the findings we have reported previously from a larger longitudinal study using same method [32]. It is plausible, that in a hemodynamically stable pregnant woman who is not volume depleted and has normal cardiac inotropy, the increasing oxygen demand by the fetus and placenta with advancing gestation is met by an increase in heart rate to maintain a stable CI.

It is conceivable that natriuretic peptides contribute to the regulation of fluid balance, hemodynamics, and cardiac function during pregnancy. However, the relation between natriuretic peptides and maternal cardiovascular hemodynamics has been investigated by only a few studies [12, 13, 19, 33]. In general, more data are available on BNP/NT-proBNP compared to ANP and CNP both in normal and complicated pregnancy. However, all three natriuretic peptides seem to have quite similar physiological trend with relatively stable levels during pregnancy and clinically significant increase only seen in pathological states. A more recently published longitudinal study of BNP/NT-proBNP also confirms this physiological trend [16]. ANP is known to regulate and reduce intravascular circulating volume and blood pressure by inducing natriuresis and inhibiting aldosterone production [2]. Slight but significantly decreasing trend of proANP with increasing gestation suggests that it might be one of the mechanisms of maintaining increased blood volume during physiological pregnancy and increasing or high proANP levels may indicate maternal cardiovascular maladaptation to pregnancy. As the ANP is shown be increased in preeclampsia, a condition characterized by reduced plasma volume, mechanism other than intravascular volume load may play a dominant role in ANP release under such conditions. A slight decrease in proANP towards the third trimester may also be associated with relatively increased compression of inferior vena cava by the gravid uterus in the supine position with advancing gestation [12]. However, we consistently performed venous blood sampling only after women have rested for 15 min in a semi-recumbent position to avoid the effect of inferior vena cava compression.

Atrial stretch is the main mechanism of stimulating ANP production, although several other factors are also involved [34]. In concordance with previous studies (Castro et al., 1992), we found maternal proANP levels to be slightly higher than the values observed in healthy nonpregnant women, suggesting that the production might be enhanced during pregnancy considering the dilution effect increased plasma volume in pregnancy. Both fetus and mother are known to independently secret and metabolize ANP. However, fetal contribution to maternal proANP levels is unlikely as only mature form of ANP has been detected in the fetal circulation [35].

Maternal CNP levels are lower in pregnant women compared to non-pregnant women, and levels are reported to fall with advancing gestation in normal pregnancies [17, 18], although a recent study by Kuessel et al. showed NT-proCNP to decrease in the first and second trimester reaching a nadir at approximately 26 weeks and increase again in the third trimester [36]. This discrepancy might be related to differences in the peptide measured (CNP vs. NT-proCNP). Our longitudinal study confirmed that the maternal plasma CNP levels decease significantly with gestational age, although the magnitude of reduction was quite small.

The CNP is known to influence both cardiac and vascular function, and it regulates vascular tone, reactivity and microvascular pressure [8, 37]. CNP promotes endothelial cell proliferation and nitric oxide synthetase activity in endothelial cells. Prior studies in men and non-pregnant women have shown an association between CNP levels and arterial stiffness/elastance and NO-dependent FMD [38, 39]. However, our study, first to investigate the association between plasma CNP and maternal endothelial function as well as systemic hemodynamics, did not reveal any significant correlation between CNP and brachial artery FMD% (a gold standard measure of endothelial function) or systemic hemodynamics including SVRI, which is considered as an indirect measure of vascular tone and endothelial function [40]. The SVRI increased significantly and the brachial artery FMD% did not change significantly with gestational age during 22–40 weeks, suggesting that nitric oxide dependent recruitable capacity of the endothelium in response to reactive hyperemia is attenuated in the second half of pregnancy. No increase in FMD% in the second half of pregnancy is not surprising as the baseline brachial artery, which is known to affect its vascular reactivity, increased significantly with gestational age. It is known that larger the diameter the smaller is the percentage change following flow-mediated shear stress [28], and the baseline diameter increased significantly with gestational age. However, as the FMD is mainly dependent on the coupling of endothelial NO synthetase and bioavailability of NO, it does not provide any information on baseline endothelial function. Whether CNP reflects baseline endothelial function in pregnant women is not known.

Fibrinogen is an acute phase glycoprotein produced in the liver. It pays an important role in vascular homeostasis, and elevated circulating levels are known to be associated with inflammation and increased risk of thrombosis and cardiovascular disease [41]. In our study, maternal plasma fibrinogen levels were higher compared to nonpregnant reference values. We found fibrinogen to correlate with proANP but not with CNP. Whether fibrinogen is a better biomarker of vascular function in pregnancy is worth exploring.

The previous research on the relationship of natriuretic peptides with cardiac performance was mostly carried out in subjects with cardiac dysfunction. Studies in subjects without cardiac impairment are few [34] and studies in normal human pregnancy are scarce [42]. A few cross-sectional studies of limited sample size have shown that natriuretic peptides may be elevated in certain pregnancy complications, such as gestational hypertension, preeclampsia, fetal growth restriction, may predict development of such complications [33, 43–46], or have a prognostic value [42]. In our study population of healthy pregnant women, plasma proANP and CNP levels did not correlate significantly with cardiovascular function. Therefore, whether these natriuretic peptides can be used as reliable biomarkers of early-stage subtle cardiovascular dysfunction during pregnancy remains doubtful.

Pregnancy is associated with marked alteration in renal perfusion and function [47]. However, an accurate method of renal function assessment in pregnancy has yet to be established. GFR by inulin clearance is the accepted standard measure of renal function, but it is cumbersome, costly, and less suitable to measure during pregnancy. Therefore, measurements of plasma uric acid, creatinine and cystatin C are commonly used in clinical practice. However, the reported normal values during pregnancy vary between cohorts, and reliable reference ranges are still lacking [48]. We provide longitudinal reference intervals for the second half of pregnancy with reasonable number of observations per gestational week. The GFR can be estimated from creatinine and cystatin C values using several different formulae, but they are poorly validated in pregnant population. A previous study on 12 pregnant women both cystatin C based, and creatinine based GFR did not correlate significantly with inulin clearance [49]. However, as creatinine based eGFR is shown to be inaccurate during pregnancy [50, 51], and cystatin C is less influenced by body mass and is a better predictor of outcomes [52], we used cystatin C to calculate eGFR using three different equations. Not surprisingly, the values obtained were different using different equations, but the temporal relation with gestational age was similar. Our values were close to that reported by another longitudinal study of 52 normal pregnancies using one of the same formulae to calculate the cystatin C based eGFR that showed a declining trend with gestation and median values below 120 mL/min/1.73 m^2^ in the third trimester of pregnancy [53]. In our study, proANP and CNP did not correlatee with any of the parameters of renal function including eGFR.

### Strengths and limitations

Assessment of cardiovascular function can be performed by invasive and non-invasive methods, although latter, are preferred methods during pregnancy. We used ICG which is a simple, safe, noninvasive validated technique well-suited for longitudinal monitoring of maternal cardiac function and systemic hemodynamics both in ambulatory and hospital settings [32, 54, 55]. Similarly, we used brachial artery FMD, a noninvasive gold standard method for evaluating endothelial function. To our knowledge, the association between maternal natriuretic peptide (proANP and CNP) levels and parameters of hemodynamic, vascular endothelial and renal function has not been evaluated longitudinally in previously published studies.

Our study has some limitations. Lack of nonpregnant controls is a major limitation. Our observations are valid and applicable only for the second half of pregnancy, since the participants were not examined before pregnancy, during the first half of the pregnancy and postpartum. Therefore, longitudinal studies including the first trimester of pregnancy and preferably also the preconception and postnatal periods are needed to understand the physiological trends. What is the most acceptable form of natriuretic peptide to measure can be arguable. For a comprehensive evaluation of physiological trend, both the mature forms as well as prohormones of ANP, BNP and CNP should have been measured. However, considering limited samples, cost and resources available, and the fact that more data are published on BNP/NT-proBNP compared to other natriureric peptides in pregnancy, we chose to measure proANP and CNP. As all the study participants were White European women, our findings may not be generalizable to other ethnic groups reducing external validity. Women’s age and parity can influence cardiovascular status. However, our sample size was not large enough to perform stratified subgroup analyses.

## Conclusions

Longitudinal reference ranges were established for the maternal plasma proANP and CNP during 22–40 weeks of pregnancy. These natriuretic peptides were significantly associated with gestational age and decreased slightly with advancing gestation, but they did not reflect the temporal physiological changes in maternal systemic hemodynamics, vascular endothelial function, or renal function during the second half of pregnancy. The proANP correlated with the thoracic fluid content reflecting volume load in pregnancy. ProANP and CNP should not be used as a direct representation of cardiovascular function in normal pregnancy, although abnormal levels may indicate maladaptation and increased risk of complications.

## Data Availability

The dataset generated and analyzed in the current study is not publicly available. However, anonymized data can be made available from the corresponding author on reasonable request.

## Abbreviations

ACI: Acceleration index
ANP: Atrial natriuretic peptide
CI: Cardiac index
CNP: C-type natriuretic peptide
eGFR: Estimated glomerular filtration rate
FMD: Flow mediated vasodilation
HR: Heart rate
LVET: Left-ventricular ejection time
MAP: Mean arterial pressure
PEP: Pre-ejection period
Q_BA_: Brachial artery volume blood flow
SVR: Systemic vascular resistance
SVRI: Systemic vascular resistance index
SV: Stroke volume
STR: Systolic time ratio
TAMxV: Time-averaged maximum velocity
TFC: Thoracic fluid content
VI: Velocity index
